# Lifelong changes of neurotransmitter receptor expression and debilitation of hippocampal synaptic plasticity following early postnatal blindness

**DOI:** 10.1038/s41598-022-13127-y

**Published:** 2022-06-01

**Authors:** Hardy Hagena, Mirko Feldmann, Denise Manahan-Vaughan

**Affiliations:** 1 Medical Faculty, Department of Neurophysiology, Ruhr University Bochum, Universitätsstr. 150, MA 4/150, 44780 Bochum, Germany; 2 International Graduate School of Neuroscience, Ruhr University Bochum, 44780 Bochum, Germany

**Keywords:** Long-term potentiation, Sensory processing

## Abstract

In the weeks immediately after onset of sensory loss, extensive reorganization of both the cortex and hippocampus occurs. Two fundamental characteristics comprise widespread changes in the relative expression of GABA and glutamate receptors and debilitation of hippocampal synaptic plasticity. Here, we explored whether recovery from adaptive changes in the expression of plasticity-related neurotransmitter receptors and hippocampal synaptic plasticity occurs in the time-period of up to 12 months after onset of sensory loss. We compared receptor expression in CBA/J mice that develop hereditary blindness, with CBA/CaOlaHsd mice that have intact vision and no deficits in other sensory modalities throughout adulthood. GluN1-subunit expression was reduced and the GluN2A:GluN2B ratio was persistently altered in cortex and hippocampus. GABA-receptor expression was decreased and metabotropic glutamate receptor expression was altered. Hippocampal synaptic plasticity was persistently compromised in vivo. But although LTP in blind mice was chronically impaired throughout adulthood, a recovery of the early phase of LTP became apparent when the animals reached 12 months of age. These data show that cortical and hippocampal adaptation to early postnatal blindness progresses into advanced adulthood and is a process that compromises hippocampal function. A partial recovery of hippocampal synaptic plasticity emerges in advanced adulthood, however.

## Introduction

Although the brain adapts to both progressive and complete sensory loss^1–4^, it has recently become apparent that the required cortical reorganization^5,6^ is accompanied by extensive changes in the expression of plasticity-related neurotransmitter receptors in sensory and association cortices, as well as in the hippocampus^7,8^. These changes that develop immediately after sensory loss and are still evident for several weeks are accompanied by an impairment of hippocampal long-term potentiation (LTP) and hippocampus-dependent learning^7,8^. This suggests that cortical adaptation to sensory loss comes at a price, and indicates that during adaptation, hippocampal function is compromised. Although blind individuals develop adept compensatory sensory and spatial skills^5^, age-related vision loss, such as glaucoma is accompanied by cognitive deficits^9^.

Although it is well-documented that children and young adults adapt very well to complete blindness^5,10–13^, individuals who become blind in mature or late adulthood have greater difficulty^14–17^, although crossmodal compensation occurs^18^. Thus, in general, the earlier complete sensory loss occurs, the better the ostensible functional adaptation may be^19^. Nonetheless, it has also been reported that early (transient) visual deprivation undermines the development of crossmodal information processing at the cognitive and multisensory levels^20,21^. Furthermore, the precise nature of compensatory adaptations at the level of spatial tuning^22^ or crossmodal adaptation^6^ depend on whether blindness occurs in early or late life.

There is consensus that adaptation to complete blindness, in terms of multisensory compensation, effectivity of spatial acuity and cognitive ability that occurs outside the ‘critical period’ of cortical development, is extremely effective in young healthy individuals^5,6,19^. The effects on sensory, spatial and cognitive adaptation of degressive visual input i.e. cumulative blindness, are less well documented, although some studies suggest that a correlation between visual and cognitive impairments exist^23–25^. Moreover, glaucoma, an eye-disease characterized by progressive loss of vision, is associated with cognitive impairment^9^.

Cortical adaptation to sensory loss is enabled by cortical plasticity^26–29^ and a reversion to an early postnatal plasticity state, akin to conditions extant during the critical period, is a characteristic of the initial cortical response to visual deprivation, or blindness^30–35^. This state, in turn, is enabled by an emulation of the postnatal (elevated) expression of the GluN2B subunit of the *N*-methyl-d-aspartate (NMDAR) receptor^33^ and of inhibitory control by GABA receptors^36,37^. This adaptation alters excitability levels in the cortex and facilitates the expression of cortical plasticity that underpins cortical reorganization^32,38–40^. Changes in NMDAR or GABA receptor expression that occur in the weeks after the onset of congenital blindness^7^ in rodents are consistent with the abovementioned reversion to a critical period-like state in the cortex. Surprisingly, however, during this adaptive phase hippocampal plasticity and hippocampus-dependent learning are impaired^7^. Given the abovementioned reports of effective long-term adaptation to blindness, we wondered whether neurotransmitter receptor expression changes, stabilizes or reverts to a pre-deficit state in advanced adulthood in blind mice. Furthermore, we monitored hippocampal synaptic plasticity in freely behaving mice throughout adulthood (3–12 postnatal months).

We report that receptor expression in the hippocampus and cortex remains in a perpetual state of reorganization in the period encompassing up to 12 months after the onset of sensory loss. In fact, a critical period-like state is sustained throughout adulthood as a consequence of irreversible sensory loss. Changes in LTP expression that we detected in freely behaving mice suggest that the process of cortical adaptation to early loss of vision is a dynamic process that is sustained throughout adulthood, and which  shows very late albeit incomplete, recovery. Although compensatory processes occur at the level of crossmodal sensory acuity^41^, hippocampal information encoding in the form of synaptic plasticity remains suboptimal following sensory loss, indicating that intact multimodal sensory input is a prerequisite for effective experience-dependent information storage by the hippocampus.

## Methods

### Animals

Animal experiments were approved in advance by, and conducted under licence of, the ethics committee of the government authority of the federal state of Northrhine Westphalia (NRW) (Landesamt für Naturschutz, Umweltschutz und Verbraucherschutz, NRW), and were conducted in accordance with the European Communities Council Directive of September 22nd, 2010 (2010/63/EEC). All methods were carried out in accordance with the relevant guidelines and regulations. Sample size, outcome measures, statistical measures and experimental procedures are consistent with the ARRIVE guidelines 2.0. The design of the study did not necessitate ‘blinding’ or randomisation. Male CBA/J (Charles River, Sulzfeld, Germany) and CBA/CaOlaHsd mice (Envigo Laboratories, Venray, Netherlands) were housed in a temperature- and humidity-controlled containers (Scantainer, Scanbur Technology A/S, Karlslunde, Denmark) with a constant 12-h light–dark cycle and ad libitum food and water access. The CBA/J (PDE6brd1/PDE6brd1) mouse possesses a hereditary mutation of the PDE6B gene (retinal degeneration1, rd1)^42,43^ that results in retinal destruction by 4 weeks of age^7^. The CBA/CaOlaHsd mouse strain has no reported deficits in its sensory modalities^44–46^ and exhibits superior spatial memory compared to the CBA/J strain^7,8^.

### Tissue preparation

As described earlier^7,8^, brains were removed when mice were 2, 4, 8 or 12 months old using isoflurane for inhalational anaesthesia, followed by an intraperitoneal injection with sodium pentobarbital. Transcardial perfusion was conducted with cooled, 0.2% heparinized Ringer solution (10 min), followed by 4% paraformaldehyde (PFA) in phosphate buffered saline (PBS) (10 min). Brains were then immersed in PFA for 24 h at 4 °C, and subsequently stored in 30% sucrose in PBS. Frozen sections of 30 μm were prepared with a cryostat microtome (Leica, Solms, Germany) for Nissl staining and immunohistochemistry. Slices from CBA/J and CBA/CaOlaHsd mice were processed together to minimize inter-array variations between different cohorts. Here, control and test slices underwent antibody (AB) treatment during the same immunohistochemistry (IHC) experiment to minimize variabilities in labelling that could be caused by IHC procedures during different treatment days or sessions. Furthermore, the labelling intensity of each individual slice was normalized to regions within the same slice that do not express neurotransmitter receptors^47^. To verify tissue quality and select slices for analysis on the basis of their anatomical features Nissl staining was performed^47^.

### Immunohistochemistry

GABA_A_, GABA_B_, GluN2B and mGlu5 immunostainings were conducted using an avidin–biotin complex (ABC) method as described previously^7,48^. GluN1 subunit staining was performed by means of tyramine amplification, additionally to the ABC method, as described by others^7,49^. To evaluate expression of GluN2A, mGlu1 and mGlu2/3 subunits, we included a streptavidin enhancement^7^. Negative controls, comprising tissue incubation with separate primary and secondary antibodies, were performed in order to verify that specific binding had occurred (not shown).

For the ABC approach, free-floating sections were rinsed three times in PBS for 10 min. Afterwards, sections were placed in 0.3% H_2_O_2_ for 20 min to remove endogenous peroxidase activity to ensuring that background staining could be kept to a minimum. The sections were preincubated with blocking solution containing 20% avidin (avidin–biotin blocking kit, Vector Laboratories, Burlingame, USA), 10% normal serum (Vector Laboratories, Burlingame, USA) and 0.2% Triton X-100 (Tx) for 90 min to reduce non-specific binding. Sections were subsequently incubated overnight at room temperature after applying the AB solution, containing 20% biotin (avidin–biotin blocking kit, Vector Laboratories), 1% normal serum, 0.2% Tx and the relevant primary AB: GABA_A_ (1:400, monoclonal mouse AB, MAB341, Merck Millipore, Billerica, USA), GABA_B_ (1:500, monoclonal mouse AB, ab55051, Abcam, Cambridge, UK), GluN2B (polyclonal goat AB, sc-1469, Santa Cruz Biotechnology, Santa Cruz, USA) or mGlu5 (polyclonal rabbit AB, ab5675, Merck Millipore). The secondary AB was then applied for 90 min. For GABA_A_ and GABA_B_, a biotinylated horse-anti-mouse AB was used (1:500, BA-2001, Vector Laboratories, Burlingame, USA) For GluN2B, a biotinylated horse-anti-goat AB was used (1:500, BA-9500, Vector Laboratories, Burlingame, USA). Subsequently, sections were immersed in 1:1000 ABC-Elite detection system (Vector Laboratories), 1% normal serum and 0.1% Tx for 90 min. The staining reaction was then performed using 3,3′-diaminobenzidin (DAB, Sigma-Aldrich, St. Louis, USA) in 0.01% hydrogen peroxide PBS for 10 min^7,8^.

Where the ABC method included tyramine amplification, Tris (hydroxymethyl)-aminomethan buffered saline (TBS) was used instead of PBS, and bovine serum albumin (BSA, Sigma Aldrich, St. Louis, USA) was used instead of n-serum. The protocol was the same as that described above for GABA and GluN2B assessment, with the exception that the primary AB incubation specific to GluN1 (monoclonal rabbit AB, 1:200, AB9864R, Merck Millipore) was extended to 5 days at 4 °C. A biotinylated goat-anti-rabbit AB (1:500, BA-1000, Vector Laboratories, Burlingame, USA) was used as secondary AB. Tyramine amplification was performed as described by Adams^49^ and followed by DAB-staining.

GluN2A, mGlu1 and mGlu2/3 subunits were detected by means of streptavidin enhancement as described before^7,8^. TBS and normal serum were used as dilution medium. The initial steps in the protocol were the same as described above. Incubation with the primary AB specific to GluN2A (1:250, polyclonal rabbit AB, sc-9056, Santa Cruz Biotechnology, Santa Cruz, USA), mGlu1 (1:200, polyclonal rabbit AB, ab82211, Abcam) or mGlu2/3 (1:250, polyclonal rabbit AB, ab1553, Merck Millipore) lasted for 24 h at room temperature, and was followed by secondary AB incubation with biotinylated goat-anti-rabbit AB (1:500, BA-1000, Vector Laboratories, Burlingame, USA). The sections were then incubated with 1:1000 streptavidin (Cy™3-conjugated Streptavidin, Jackson Laboratories, Bar Harbor, Maine, USA) for 30 min. Anti-streptavidin AB (1:500, biotinylated goat-anti-streptavidin, BA-0500, Vector Laboratories, Burlingame, USA) was applied for another 30 min, followed by DAB staining.

**Figure 1:**
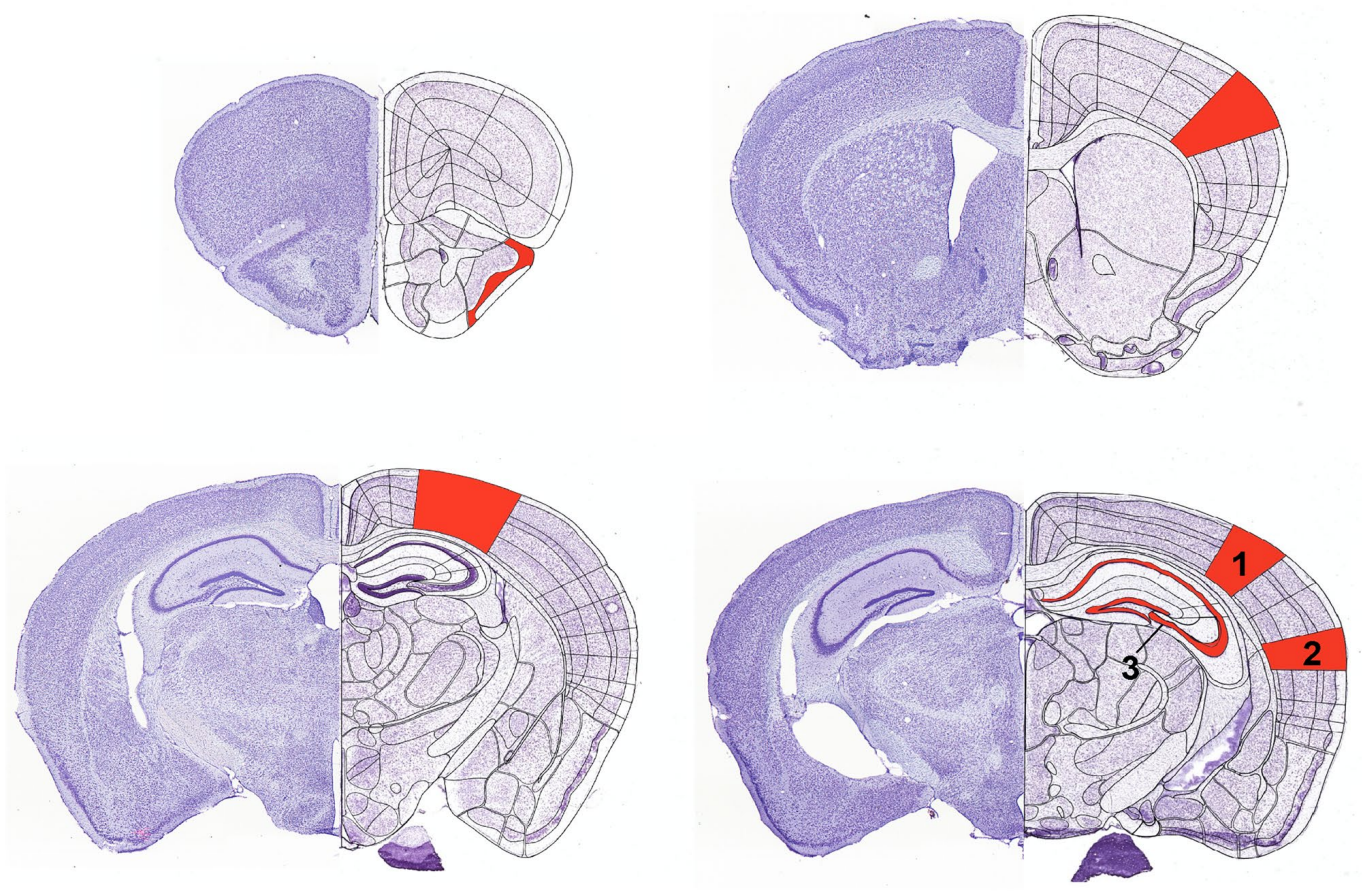
Diagram of areas selected for immunohistochemical analysis. The areas assessed in the mouse cerebral cortex and hippocampus are shown in the cresyl violet-stained histological sections (left side of each example). The specific regions examined are indicated in the schemas on the right side of each example (modified from the Allen Mouse Brain Atlas (mouse.brain-map.org) and the Allen Reference Atlas—Mouse Brain (atlas.brain-map.org)^50–55^). Markings in red represent the following areas: Top left: piriform cortex. Top right: somatosensory cortex. Bottom left: posterior parietal cortex. Bottom right: visual cortex (1), auditory cortex (2) and hippocampus (3) including the dentate gyrus, and cornus ammonis subregions (CA1–CA3).

Brain sections from CBA/J mice were examined for changes in the expression of GABA_A_ and GABA_B_ receptors, mGlu1, mGlu2/3 and mGlu5 receptors and the GluN1, GluN2A and GluN2B subunits of the NMDAR compared to equivalent sections taken from CBA/CaOlaHsd mice. The following cortical regions were scrutinized: Piriform cortex (PiC), somatosensory cortex (SC), posterior parietal cortex (PPtA), visual cortex (VC), auditory cortex (AuC). In addition, the following regions of the dorsal hippocampus were examined: dentate gyrus (DG), CA1 region, CA3 region and CA4 region (Fig. 1). Some receptor expression outcomes for the 2 and 4 month- old data sets were reported previously^7^ and these are summarised in Supplementary Table S2.

### In vivo electrophysiology

All mice had reached a minimum weight of 22 g before undergoing surgical electrode implantation. Animals were anesthetized with sodium pentobarbital (60 mg/kg i.p.) and underwent stereotaxic chronic implantation of a bipolar stimulation electrode and a monopolar recording electrode as described earlier^56^. The stimulation electrode was implanted in the Schaffer collateral pathway of the dorsal hippocampus − 2.0 mm anterioposterior (AP), 2.0 mm mediolateral (ML) from bregma and ~ 1.4 mm dorsoventral (DV) from brain surface. The recording electrode was implanted in the ipsilateral CA1 stratum radiatum (AP: − 1.9 mm, ML: 1.4 mm, DV ~ 1.2 mm) and was used to monitor the evoked potentials at SC-CA1 synapses during the implantation procedure. The coordinates used for the electrodes were based on a mouse brain atlas^57^. Test-pulse recordings during surgery were used to adjust for correct depth of the electrodes. The stimulation and recording electrodes were made of polyurethane-coated stainless steel wire (100 µm diameter; Gündel, BioMedical Instruments, Germany) and were lowered into the brain through a single hole (~ 1.6 mm in diameter) that was drilled through the cranial bone. On the contralateral side, two additional holes (~ 0.7 mm in diameter) were drilled though the bone into which two anchor screws were inserted. Stainless steel wires (A–M Systems) were attached to the screws, serving as reference and grounding electrodes, respectively. The five wires were secured to a six-pin socket (Conrad Electronic SE, Germany) and the whole assembly was fixed on the skull using dental acrylic (J. Morita Europe GmbH, Germany; Haræus Kulzer GmbH, Germany). The animals were allowed at least 10 days for recovery before experiments were commenced. During this period, animals were monitored closely for infections or distress and were handled regularly. Twenty-four hours prior to start of each experiment, animals were transferred from their housing cages to the recording chamber [40 cm (length) × 40 cm (width) × 40 cm (height)] with full access to food and water, to ensure adequate familiarization of the environment.

The socket was connected via a flexible cable that was attached via a swivel connector to the recording/stimulation system and allowed free movement of the mouse. Field excitatory postsynaptic potentials (fEPSPs) obtained in the CA1 region were used to measure changes in synaptic responses that were evoked in the stratum radiatum by stimulation of Schaffer collaterals at low frequency (0.025 Hz) with single biphasic square waves of 0.2 ms duration per half-wave by a constant current isolation unit (World Precision Instruments). The fEPSP signal was amplified using a differential alternating current (AC) amplifier (A-M Systems) and digitized through a data acquisition unit (Cambridge Electronic Design, UK). An input–output (stimulus–response) curve (stimulation intensity of 20, 30, 40, 50, 75, 100, 125 and 150 µA with a 5-min interval) was determined prior to commencing the experiment. A comparison of input–output curves between CBA/CaOlaHsd and CBA/J mice showed no significant differences at all ages that were tested (Supplementary Fig. S1 and Supplementary Table S1). Test-pulse and plasticity-inducing stimulation in all experiments were conducted using the stimulation intensity that produced an fEPSP that was 40% of the maximum fEPSP obtained in the input–output curve assessment.

### Data analysis

Optical density measurements were conducted for selected brain regions using an image-based analysis system (Neurolucida, MBF Bioscience, Williston, USA) for analysis of neurotransmitter receptor distribution. Protein quantification was conducted by means of a standardized immunoassay^7,58^. The following distances from bregma were chosen for examination of cortical and hippocampal areas: 2.2 mm (piriform cortex, Fig. 1), 0.5 mm (somatosensory cortex, Fig. 1), − 1.8 mm (posterior parietal cortex, Fig. 1), − 2.5 mm (auditory cortex, visual cortex, dentate gyrus, CA1-3, Fig. 1). Tissue sections were analysed with 2.5-fold magnification using a brightfield microscope (Leitz Wetzlar Dialux 20, Leica, Solms, Germany). Whole slide images were acquired with a digital camera (QIC-F-CLR-12, QImaging, Surrey, Canada), using the virtual tissue 2D module of Neurolucida. Immunohistochemical background staining was corrected by subtracting density values obtained in the corpus callosum to minimize inter-array differences^59^. Luminance information ranging from 0 to 255 was determined for the whole area^8^.

Immunohistochemical data were assessed for between-group effects by means of multifactorial analysis of variance (ANOVA) followed by a post-hoc Duncan’s test. Between-group factors comprised strain (e.g. CBA/J vs. CBA/CaOlaHsd) and brain area (piriform cortex: PiC, somatosensory cortex: SC, posterior parietal cortex: PPtA, visual cortex: VC, auditory cortex: AuC, dentate gyrus: DG, CA1, CA2/3, CA4) were assessed 2, 4, 8 and 12 months postnatally.

For in vivo electrophysiology, each time-point that was measured, consisted of the average of five consecutively evoked fEPSPs responses at 40 s intervals. The first six time points were recorded at 5-min intervals and served as a baseline reference. These six time points were averaged and all time points throughout the experiment were expressed as the mean percentage ± standard error of the mean (SEM) of this value. Five minutes after the sixth time point, plasticity-inducing electrical stimulation (high-frequency stimulation, HFS) was applied. The first three time points after HFS were recorded with a 5 min interval and then every 15 min until 4 h had elapsed. To determine the longevity of any changes in synaptic plasticity, a further 1 h recording was performed the next day, approximately 24 h after the experiment began. Changes in synaptic transmission were determined by measuring the slope obtained on the first negative deflection of the evoked fEPSP. The plasticity-inducing stimulation consisted of 4 times 50 pulses at 100 Hz^60^. Mice that showed no adequate electrophysiological responses after implantation were discarded from the study. No behavioral changes were observed during the experiment. The data presented are from the same n = 17 (n = 9 CBA/J and n = 8 CBA/CaOlaHsd) mice throughout the study. The same animals were tested at 3, 4, 5, 6, 9, 10, 11 and 12 months of age. Animals therefore served as their own controls in the respective experiments.

Between-group effects in electrophysiological data were assessed by means of a two-way ANOVA with repeated measures. A post hoc Fisher`s test was used to discriminate significant effects at specific time-points/conditions. In the subsequent text sections, the number of animals is signified by an upper case, ‘N’, the number of slices used, is signified by a lower case ‘n’.

All data were shown as mean ± standard error of mean. All statistical tests were performed using STATISTICA 12 (Statsoft). The level of significance was set at p < 0.05.

## Results

### In mature adulthood, the expression of the GluN1 subunit of the NMDA receptor in blind mice is decreased in all sensory cortices, in the posterior parietal cortex and in the hippocampal subfields

**Figure 2:**
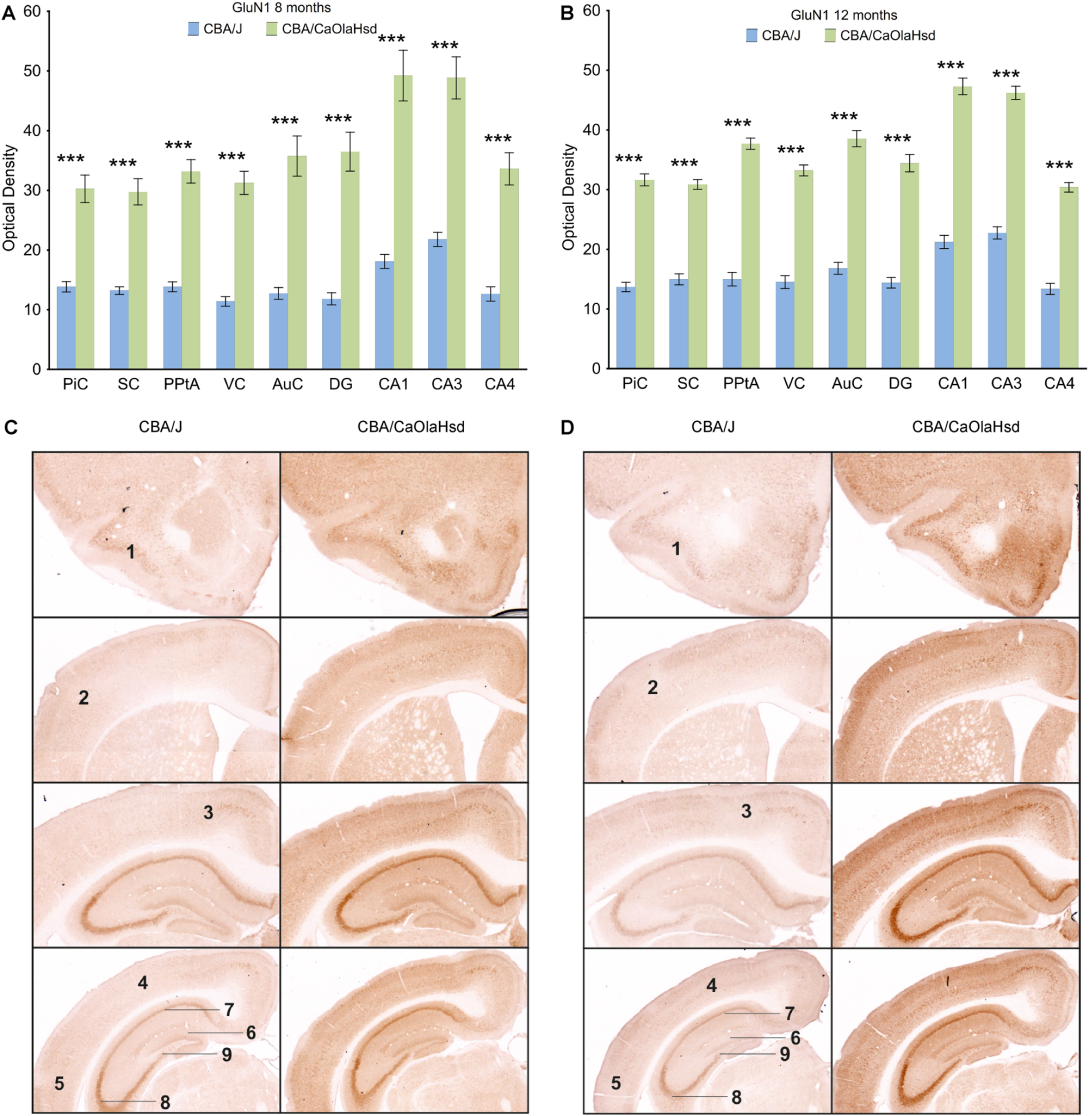
GluN1 expression is globally decreased in blind mice 8 months and 12 months postnatally. (**A**, **B**) Bar charts represent mean GluN1 subunit optical densities in different brain regions 8 and 12 months postnatally in CBA/J (blind) mice compared with CBA/CaOlaHsd mice. Subunit density is significantly decreased in the piriform cortex (PiC), somatosensory cortex (SC), posterior parietal cortex (PPtA), visual cortex (VC), auditory cortex (AuC), dentate gyrus (DG), CA1, CA3, and CA4 in CBA/J mice. *P < 0.05; **P < 0.01; ***P < 0.001. (C,D) DAB-stained coronary sections showing GluN1 expression in CBA/J and CBA/ CaOlaHsd mice 8 and 12 months postnatally. The photomicrographs highlight the changes as described in (**A** and **B**). 1: piriform cortex, 2: somatosensory cortex, 3: posterior parietal cortex, 4: visual cortex, 5: auditory cortex, 6: dentate gyrus, 7: CA1, 8: CA3, 9: CA4.

To investigate to what extent early onset blindness has on long-term effects on cortical and hippocampal function, we used immunohistochemical analysis to evaluate receptor expression in CBA/J mice (n = 6), and CBA/CaOlaHsd (n = 6). Given their essential role in NMDAR function we scrutinized expression of the GluN1 subunit of the NMDAR. No changes in subunit expression were detected at 2 and 4 months of age compared to CBA/CaOlaHsd mice (Supplementary Table S2). In CBA/J mice, at 8 and 12 months of age, we detected a significant loss of this subunit in all areas scrutinized. These comprised the piriform, somatosensory, parietal, visual, auditory cortices and all subfields of the hippocampus (CA1-4, DG) (Fig. 2, Supplementary Table S2).

### GluN2A subunits decrease in blind mice at 8 and 12 months of age

**Figure 3:**
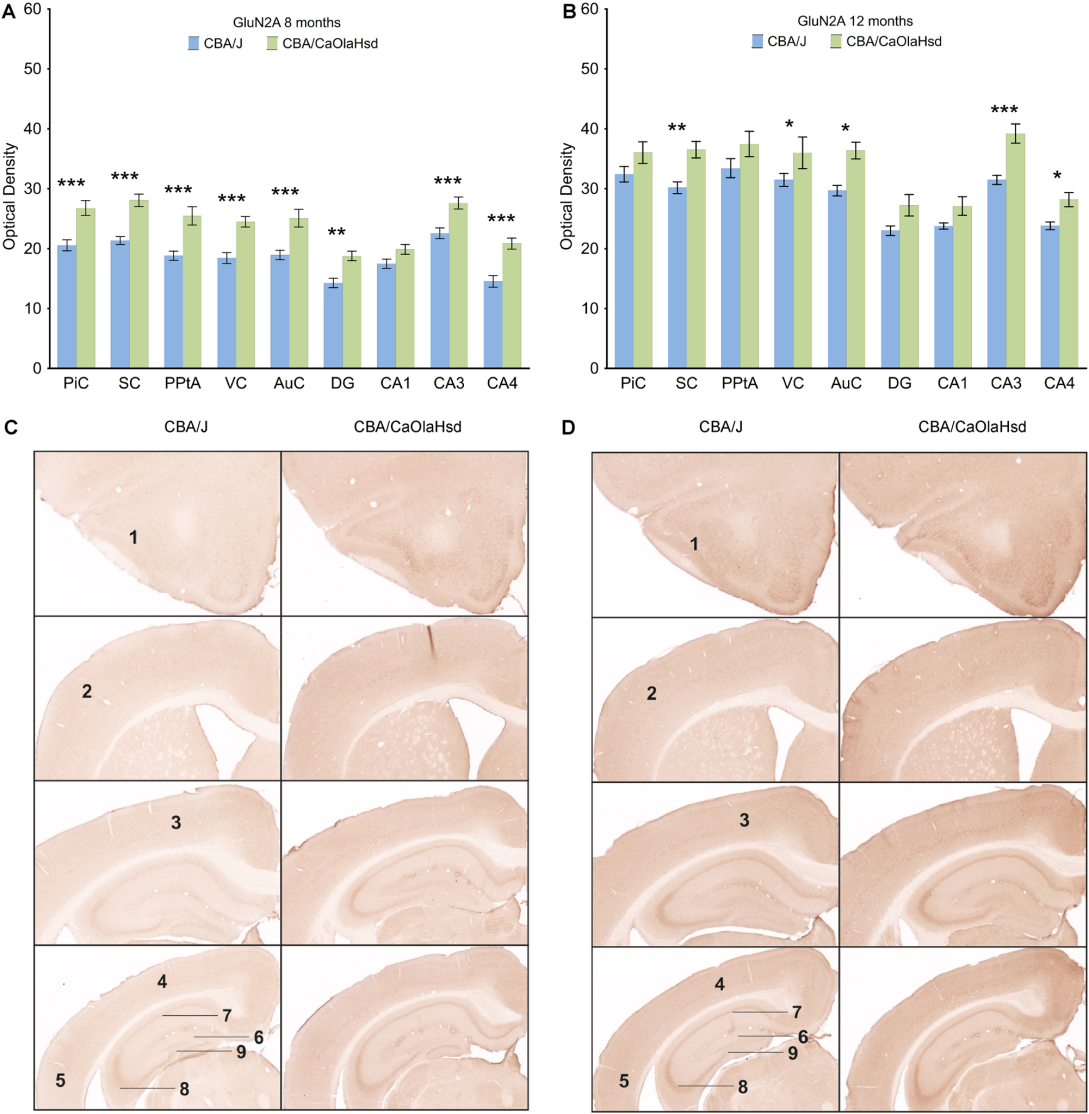
GluN2A expression is reduced in blind mice 8 months and 12 months postnatally compared to CBA/CaOlaHsd mice. (**A**, **B**) Bar charts represent mean GluN2A subunit optical densities in different brain regions 8 and 12 months postnatally in CBA/J (blind) mice compared with CBA/CaOlaHsd mice. Except in CA1, subunit density is significantly decreased in the piriform cortex (PiC), somatosensory cortex (SC), posterior parietal cortex (PPtA), visual cortex (VC), auditory cortex (AuC), dentate gyrus (DG), CA3, and CA4 in CBA/J mice 8 months postnatally. 12 months after birth, subunit density is still reduced in somatosensory cortex (SC), visual cortex (VC), auditory cortex (AuC), CA3 and CA4 in CBA/J mice. *P < 0.05; **P < 0.01; ***P < 0.001. (**C**, **D**) DAB-stained coronary sections showing GluN2A expression in CBA/J and CBA/CaOlaHsd mice 8 and 12 months postnatally. The photomicrographs highlight the changes as described in (**A** and **B**). 1: piriform cortex, 2: somatosensory cortex, 3: posterior parietal cortex, 4: visual cortex, 5: auditory cortex, 6: dentate gyrus, 7: CA1, 8: CA3, 9: CA4.

We then examined the expression of the GluN2A subunit of the NMDAR. Here, a more localized changes in expression patterns emerged. In 8 month old blind mice, subunit expression was significantly reduced in all cortical and hippocampal areas studied, with the exception of the CA1 region (Fig. 3A,C; n = 6). In 12 month-old CBA/J mice, subunit expression was still reduced in the somatosensory, auditory and visual cortices, as well as in the CA3 and CA4 regions (Fig. 3B,D; Supplementary Tables S2 and S3).

### GluN2B subunits decrease in 12 month-old blind mice

**Figure 4:**
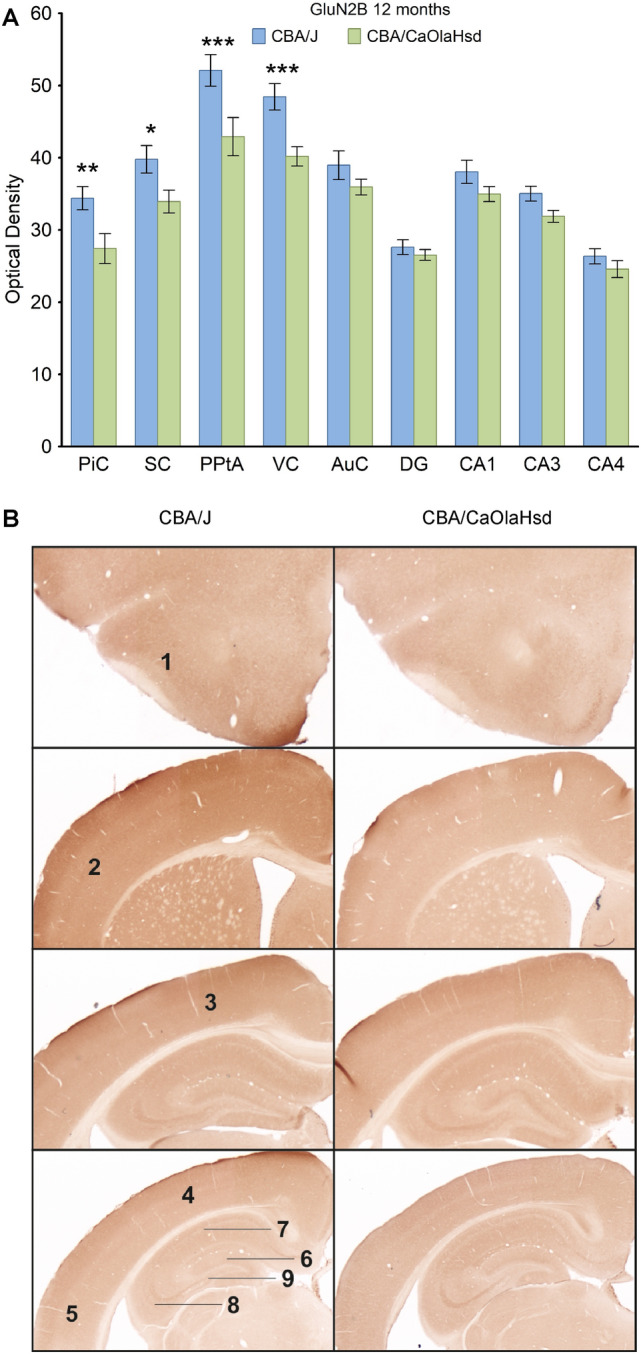
GluN2B expression is partially increased in blind mice 12 months postnatally compared to CBA/CaOlaHsd mice. (**A**) Bar charts represent mean GluN2B subunit optical densities in different brain regions 12 months postnatally in CBA/J (blind) mice compared to CBA/CaOlaHsd mice. Subunit density is significantly increased only in the piriform cortex (PiC), somatosensory cortex (SC), posterior parietal cortex (PPtA) and visual cortex (VC) in CBA/J mice. *P < 0.05; **P < 0.01; ***P < 0.001. (**B**) DAB-stained coronary sections showing GluN2B expression in CBA/J compared to CBA/CaOlaHsd mice 12 months postnatally. The photomicrographs highlight the changes as described in (**A**). 1: piriform cortex, 2: somatosensory cortex, 3: posterior parietal cortex, 4: visual cortex, 5: auditory cortex, 6: dentate gyrus, 7: CA1, 8: CA3, 9: CA4.

When we examined GluN2B subunit expression, we found no changes in CBA/J (n = 6) mice at 8 months of age (Supplementary Tables S2 and S3) compared to controls, although wide-ranging cortical and hippocampal increases of expression occurred at 4 months of age (Supplementary Table S3). By 12 months, expression was increased in the piriform, somatosensory, parietal and visual cortices, but not the hippocampus of CBA/J mice (Fig. 4A,B; supplementary Tables S2 and S3).

### Metabotropic glutamate receptor expression exhibits localised changes in blind mice

We then scrutinized expression of mGlu1, mGlu2/3 and mGlu5 receptors in the hippocampus and cortex. Here, we detected isolated increases in mGlu1 receptor expression in 4 month old, but not 2 month-old, blind mice compared to controls (Supplementary Table S2). We also detected a decrease in mGlu1 receptor expression of 8 month-old CBA/J mice in the piriform, somatosensory, visual and auditory cortices (Supplementary Tables S2 and S3), but not in the hippocampus. At 12 months, expression was increased in the parietal, and visual cortices, as well as in all subfields of the hippocampus (Supplementary Tables S2 and S3).

Expression of mGlu2 receptors also exhibited a similar punctate pattern, as well as a time-dependency of expression patterns. Localized increases in receptor expression occurred at 2 and 4 months in blind mice (Supplementary Table S3). By contrast, in 8 month-old CBA/J mice, expression was decreased in all regions excepting the piriform cortex, DG and CA1 region (Supplementary Tables S2 and S3). In 12 month-old CBA/J mice, no changes in expression were evident.

Expression of mGlu5 receptors was unchanged in 2 and 4 month-old CBA/J mice (Supplementary Table S2). Expression was also unchanged in 8 and 12 month old CBA/J mice compared to controls (Supplementary Tables S2 and S3).

### GABA_A_ and GABA_B_ expression shows localized decreases in blind mice

**Figure 5:**
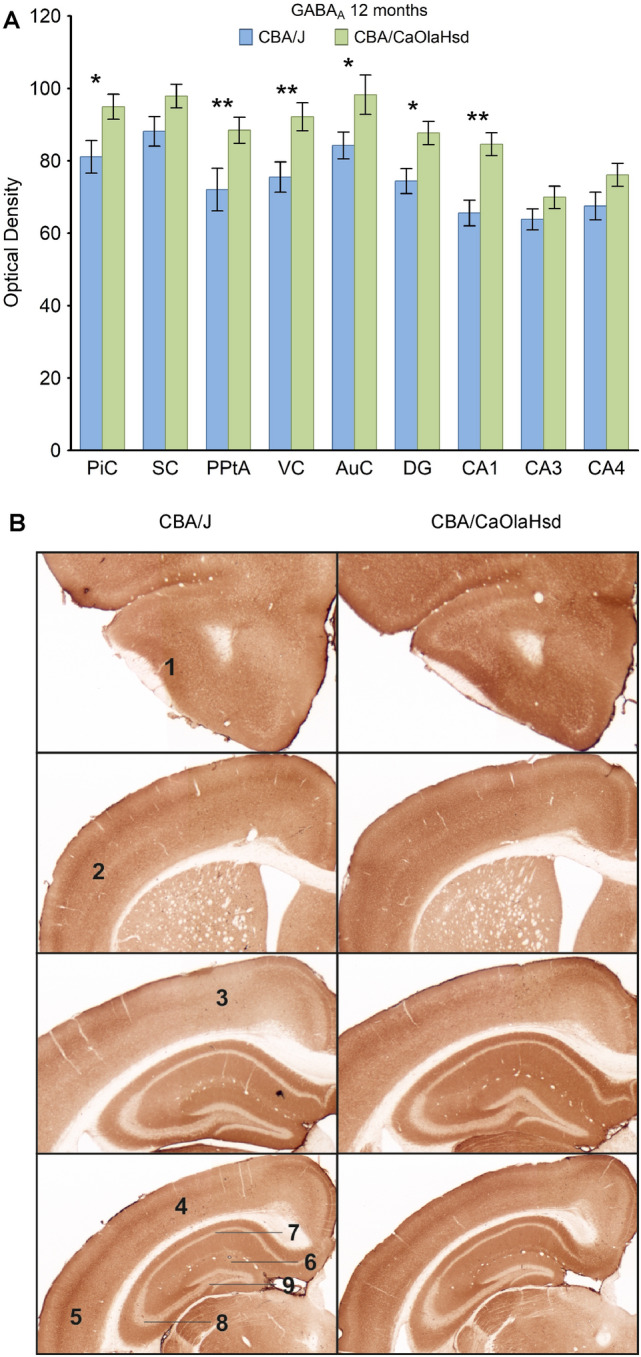
GABA_A_ expression is differentially expressed in blind mice compared to CBA/CaOlaHsd mice 12 months postnatally. (**A**) Bar charts represent mean GABA_A_ receptor optical densities in different brain regions 12 months postnatally in CBA/J (blind) compared to CBA/CaOlaHsd mice. Receptor density is significantly reduced in all areas except CA3 and CA4 in CBA/J mice. *P < 0.05; **P < 0.01; ***P < 0.001. (**B**) DAB-stained coronary sections showing GABA_A_ expression in CBA/J compared to CBA/CaOlaHsd mice 12 months postnatally. The photomicrographs highlight the changes as described in (**A**). 1: piriform cortex, 2: somatosensory cortex, 3: posterior parietal cortex, 4: visual cortex, 5: auditory cortex, 6: dentate gyrus, 7: CA1, 8: CA3, 9: CA4.

GABA_A_ expression was reduced solely in the CA1 region of 8 month-old CBA/J mice n = 6), and was reduced in all areas studied with the exception of the somatosensory cortex, CA3 and CA4 regions in 12 month old animals (Fig. 5A,B; supplementary Tables S2 and S3).

A similar punctate pattern of changes was evident with regard to GABA_B_ expression. Here, we detected decreases in the CA1 and CA3 regions of 8 month-old CBA/J mice, whereas by 12 months reductions were only evident in the CA1 region and auditory cortex (Supplementary Tables S2 and S3).

### Changes in synaptic plasticity in blind mice

It has been recently reported that LTP in the hippocampal slice preparation is impaired in CBA/J mice^7^ 4 months postnatally compared to LTP obtained in CBA/CaOlaHsd mice. Impairments are accompanied by early changes in plasticity-related neurotransmitter receptor expression in the hippocampus and cortex^7^. Given the profound and prolonged changes in receptor expression that we detected throughout adulthood in blind mice and the crucial role of sensory inputs in enabling hippocampal synaptic plasticity in vivo^61–63^, we wondered if synaptic plasticity in freely behaving mice is affected. For this we recorded evoked responses from CBA/J and CBA/CaOlaHsd mice over a period of one year beginning at three months of age. To assess LTP, we stimulated the Schaffer collaterals with 100 Hz high frequency stimulation (HFS, 4 × 50 pulses at 5 min intervals) and recorded fEPSPs from freely behaving mice for a period of over 24 h during each experiment.

### Blind mice express markedly poorer LTP compared to mice that have no sensory deficit. Impairments are consistent across the lifespan of the animals

**Figure 6:**
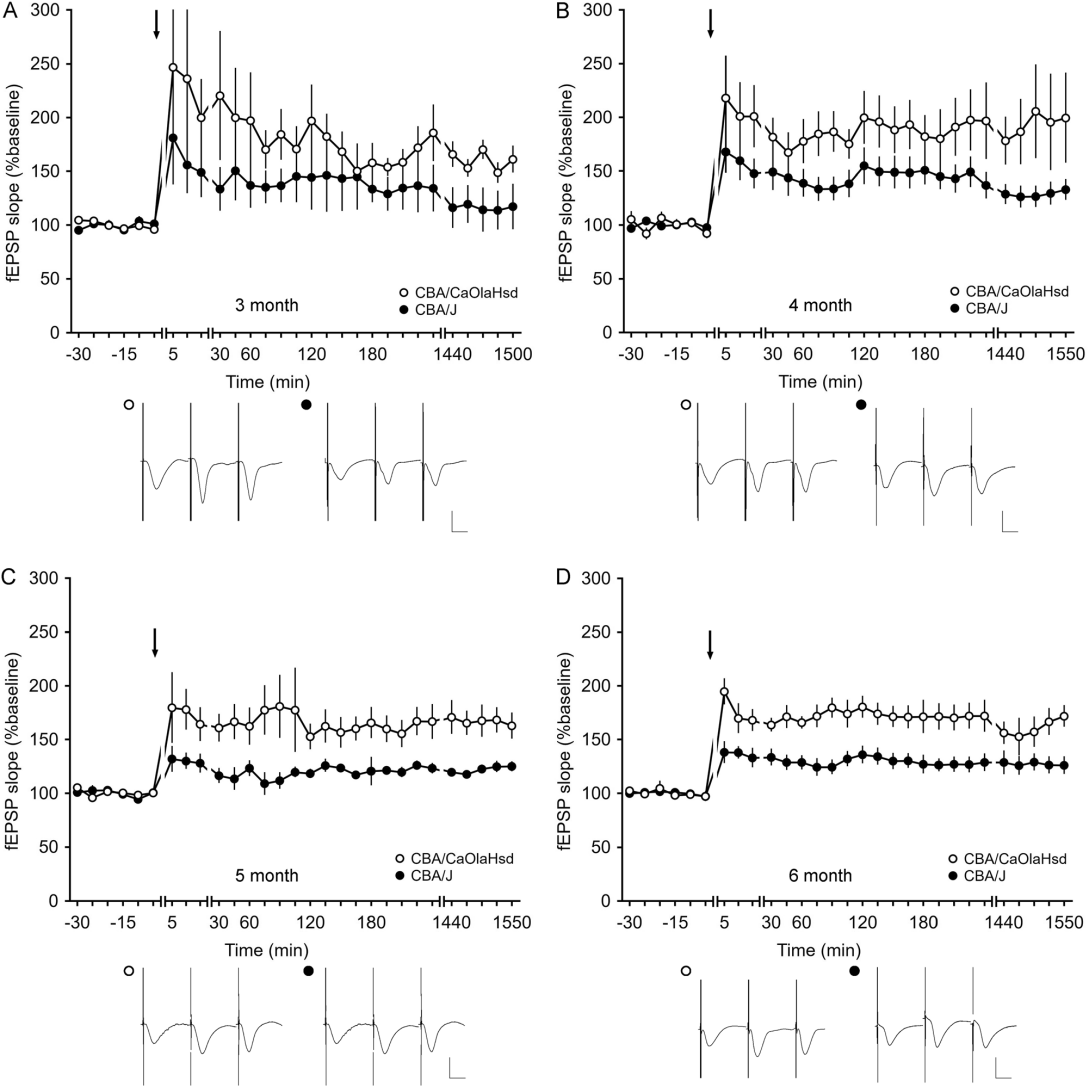
Comparison of hippocampal long-term potentiation of CBA/CaOlaHsd and CBA/J mice 3–6 months after birth. Long-term potentiation that lasted for longer than 24 h was elicited in CBA/CaOlaHsd and CBA/J mice by high frequency stimulation (HFS; 4 × 50 pulses at 100 Hz). (**A**) Three months after birth, CBA/CaOlaHsd mice expressed LTP which was not statistically different to LTP in CBA/J (blind) mice. However, 4 (**B**), 5 (**C**) and 6 (**D**) months after birth, CBA/CaOlaHsd mice showed significantly larger LTP than in CBA/J mice of the same age. Line breaks indicate change in time scale. Arrows depict the time of stimulation. Analogs represent responses before stimulation, after stimulation and 24 h after stimulation in CBA/CaOlaHsd (open circles) and CBA/J (closed circles) mice. Vertical scale bar: 2 mV; horizontal scale bar: 10 ms.

At an age of 3 months, CBA/CaOlaHsd mice responded to HFS with long-term potentiation (LTP) that lasted for over 24 h (Fig. 6A). CBA/J mice responded to the same stimulation paradigm with LTP of smaller magnitude that was however not statistically different compared to LTP in CBA/CaOlaHsd mice (ANOVA, *F*_1,11_ = 1.74, *p* = 0.21; interaction effect*: F*_*22*,242_ = 0.52, *p* = 0.97; *n* = 6 for CBA/J and n = 7 for CBA/CaOlaHsd; Fig. 6A).

Four months after birth, HFS resulted in a significantly larger LTP in CBA/CaOlaHsd mice compared to CBA/J mice (ANOVA, *F*_1,14_ = 6.70, *p* < 0.05; interaction effect*: F*_*22*,308_ = 0,55; *p* = 0.95; *n* = 9 for CBA/J and n = 7 for CBA/CaOlaHsd; Fig. 6B).

The same consistently poorer LTP response can be observed in 5 and 6 month old CBA/J mice, compared to controls: LTP in 5 month old CBA/CaOlaHsd animals show significantly higher LTP expression for over 24 h compared to CBA/J mice (ANOVA, *F*_1,9_ = 9.07, *p* < 0.05; interaction effect*: F*_*22*,198_ = 0,37; *p* = 0.99; *n* = 5 for CBA/J and n = 7 for CBA/CaOlaHsd; Fig. 6C).

As with younger animals, 6 month-old CBA/CaOlaHsd animals show LTP of a significantly greater magnitude compared to CBA/J mice (ANOVA, *F*_1,11_ = 7.09, *p* < 0.05; interaction effect*: F*_*22*,242_ = 0,66; *p* = 0.87; *n* = 7 for CBA/J and n = 6 for CBA/CaOlaHsd; Fig. 6D).

We then examined LTP responses at the age of 9–12 months to see if an adaptive recovery to blindness or deafness occurs in the hippocampus. Here, however LTP responses in the blind mice remained poorer than responses evoked in CBA/CaOlaHsd mice (n = 8).

**Figure 7:**
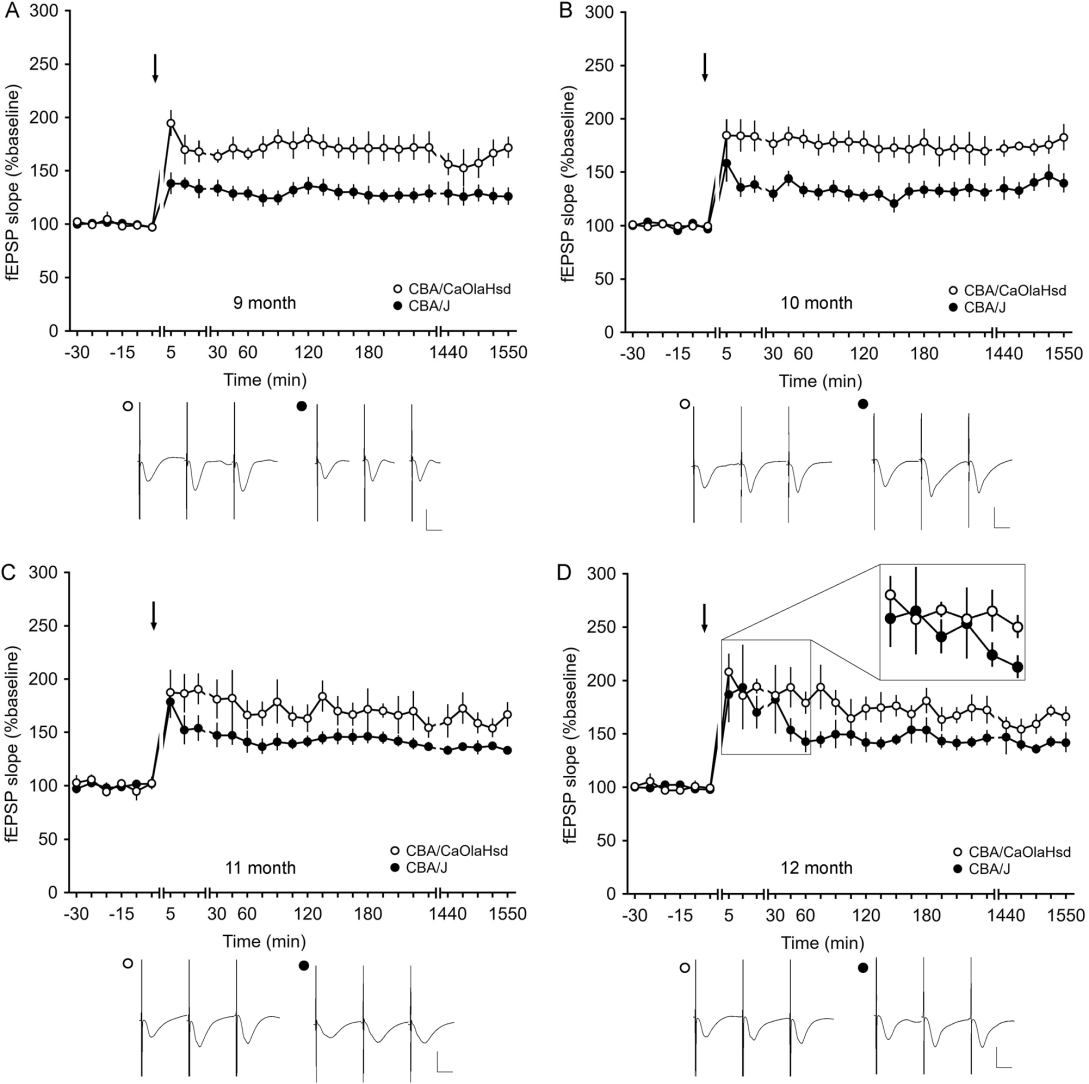
Comparison of hippocampal long-term potentiation of CBA/CaOlaHsd and CBA/J mice 9–12 months after birth. After application of HFS (4 × 50 pulses at 100 Hz), LTP was obtained in CBA/CaOlaHsd and CBA/J mice that lasted over 24 h. CBA/CaOlaHsd mice showed significantly larger LTP compared to CBA/J (blind) mice, (**A**) 9, (**B**) 10 and (**C**) 11 months after birth. (**D**) At 12 months of age, LTP elicited in CBA/CaOlaHsd mice is still more potent compared to C57BL/6 mice, although a recovery of the early component of LTP became evident. The inset shows a magnification of the first six time-points after stimulation. Line breaks indicate change in time scale. Arrows depict the time of stimulation. Analogs represent responses before stimulation, after stimulation and 24 h after stimulation in CBA/CaOlaHsd (open circle) and CBA/J (closed circle) mice. Vertical scale bar: 2 mV; horizontal scale bar: 10 ms.

In summary, 9 months after birth, LTP in CBA/J mice is significantly impaired (ANOVA, *F*_1,14_ = 14.57, *p* < 0.01; interaction effect*: F*_*22*,308_ = 0,78; *p* = 0.75; *n* = 8; Fig. 7A).

Ten month old CBA/CaOlaHsd (n = 7) mice still display significantly better plasticity compared to CBA/J mice (ANOVA, *F*_1,12_ = 9.76, *p* < 0.01; interaction effect*: F*_*22*,264_ = 0.82; *p* = 0.69; *n* = 7; Fig. 7B).

Eleven months after birth, CBA/J mice still showed impaired LTP compared to LTP evoked in CBA/CaOlaHsd mice (n = 6) (ANOVA, *F*_1,11_ = 5.37, *p* < 0.05; interaction effect*: F*_*21,*231_ = 0.43; *p* = 0.99; *n* = 7; Fig. 7C). However, by 12 months of age, a significant recovery of the early phase of LTP (5 min post-HFS) became evident in CBA/J mice compared to controls. Here, not with standing, late-LTP (> 120 min HFS) was still impaired compared to responses evoked in CBA/CaOlaHsd mice (ANOVA, *F*_1,9_ = 7.92, *p* < 0.05; interaction effect*: F*_*13*,117_ = 0.44; *p* = 0.95; *n* = 4; Fig. 7D), although early LTP was not statistically different between strains (ANOVA, *F*_1,9_ = 0.98, *p* = 0.35; interaction effect:* F*_*8*,72_ = 0.79; *p* = 0.62; *n* = 7; Fig. 7D).

Supplementary Fig. S2 shows a summary of short-term (1–3 h after HFS) and long-term (3-24 h after HFS) changes in synaptic responses between CBA/CaOlaHsd and CBA/J mice at all ages that were tested.

As the same mice were tested at the different ages, we also tested for changes in synaptic plasticity across the ages under scrutiny. CBA/CaOlaHsd mice showed no differences in the outcome of LTP when compared across the ages tested (Supplementary Table S4). In other words, LTP remained constant in magnitude and duration through the lifespan assessed. In CBA/J mice, LTP was smaller at 5 months of age compared to LTP recorded at 10 months (ANOVA, *F*_1,10_ = 5.29, *p* < 0.05; interaction effect*: F*_*22*,220_ = 0.51; *p* = 0.97), 11 (ANOVA, *F*_1,10_ = 9.10, *p* < 0.05; interaction effect*: F*_*22*,220_ = 0.95; *p* = 0.52) and 12 months (ANOVA, *F*_1,10_ = 7.0, *p* < 0.05; interaction effect*: F*_*22*,220_ = 0.97; *p* = 0.51), indicating that LTP responses in blind mice showed some degree of fluctuation throughout life (Supplementary Table S5). Taken together, however, our results show that, although LTP in CBA/J mice is impaired throughout adulthood, a recovery of the early, but not late, phase of LTP emerges at 12 months of age.

## Discussion

In this study we compared expression levels of plasticity-related neurotransmitter receptors and in vivo hippocampal synaptic plasticity from early to late adulthood in CBA/J mice that experience rapid congenital blindness. A primary finding was that the expression of the GluN1 subunit of the NMDAR was reduced in blind mice throughout adulthood, compared to sensorially intact mice, with effects being widespread in the cortex and hippocampus. Functional changes in the NMDAR were also apparent. In blind mice, GluN2A expression decreased at 8 and 12 months. GluN2B expression was increased in the cortex of blind mice at 12 months. GABA_A_ expression was unchanged in blind mice at 8 months, but decreased in multiple cortical structures and in both hippocampal CA1 and dentate gyrus (DG) in 12 month-old blind mice. GABA_B_ expression was decreased in localized structures at 8 and 12 months. Metabotropic receptor, mGlu1 was increased in some structures at 12 months in blind mice, whereas mGlu5 receptor expression was unchanged. By contrast, mGlu2/3 receptor expression was decreased at 8 months and increased at 12 months in blind mice. Thus, expression of receptors that are essential for synaptic and cortical plasticity remained in a constant flux, with no little evidence of stabilization of expression occurring in adulthood. Receptor expression effects impacted on the hippocampus, where, strikingly, LTP in freely behaving blind mice was impoverished compared to control mice from early through late adulthood. LTP in the blind mice rallied at 12 months, whereupon improvements in the early, but not late, phases of LTP became apparent compared to sensorially intact mice.

There are, however, some limitations that are accompany the use of immunohistochemistry procedures, which can confound accurate data acquisition, and should therefore be mentioned. For example, an absence of antibody specificity for different receptor locations, or cell types in the brain, could mean that subtle differences in receptor expression might go undetected^64^. Furthermore, some detergents, such as Triton-X100, may result in a structural disruption of cell membranes at higher concentrations that can lead, in turn, to a detection of epitopes across sections due to an artefactual distribution of antibodies^65^. A concentration of 0.17 mM of Triton-X100has been shown to affect cell permeability in HeLa cells, for example^65^. To minimize a putative negative effect on the structural integrity of cell membranes in our study, we used a concentration of 0.03 mM. Scrutiny of the effects of chemical compounds (e.g. in control tissue samples) and assessment of cell quality is a crucial validation step in immunohistochemistry studies. Moreover, the normalization of labeling intensity within individual slices helps circumvent variable or artefactual results^47^. These stringent measures were implemented in our experiments.

An intriguing and unexpected finding of this study is that the ‘reversion’, on a molecular level, to a critical period-like state that occurs in the initial weeks after onset of blindness^7^ is largely sustained throughout adulthood. The critical period corresponds to the first three months after birth^66^ that is characterized by higher levels of neuronal excitability, lower thresholds for the induction of synaptic plasticity^67–69^ and a sensory input-driven organisation of the sensory cortices^40,70,71^ that is a prerequisite for subsequent effective functional sensory perception and representation^66,72^. Elevated cortical levels of GluN2B subunits of the NMDA receptor and decreased expression of GABA receptors, relative to levels in the adult cortex, is a specific feature of the critical period, that becomes reinstated after prolongation of postnatal eye closure^33^ and by onset of blindness in mice^7^. In the present study we observed that the abovementioned changes in NMDA and GABA receptors perpetuate into advanced adulthood in blind mice. In other words, the cortex and hippocampus do not ultimately revert to expression levels that are characteristic of a brain in which all sensory modalities are intact. This suggests that although multisensory adaptation occurs in blindness, the cortex and hippocampus never experience a full functional recovery from sensory loss. Moreover although LTP impairments improves in advanced age in blind mice, synaptic plasticity nonetheless remains impaired, compared to sensorially intact animals.

The hippocampus is involved in both spatial memory and perception^73–78^. In congenital blindness, non-visual sensory modalities and the visuospatial cortical area are intact^1,11,66,79^ and crossmodal compensation occurs^80–82^. Thus, perceptual information from these sources can be utilized and visual input is therefore not a prerequisite for the generation of visuospatial images^83^. Differences in visuospatial representations and spatial memory become apparent however with increasing task difficulty and dimensions of space. The hippocampus has been proposed to process complex catenations of spatial features^84^ and the posterior hippocampus in particular is responsible for the processing of allocentric memory^85^. In line with this, experienced taxi drivers that had acquired a complete and detailed allocentric memory of the layout of the city of London were reported to have greater gray matter density in their posterior hippocampus and less gray matter density in their anterior hippocampus compared to control subjects^86,87^. Opposite changes in hippocampal size were identified in blind individuals: the posterior hippocampus was smaller and the anterior hippocampus was larger than in sighted individuals^88^. Thus, differences in the size of the posterior hippocampus in blind individuals suggest that they may be less effective in storing representations of allocentric space.

This interpretation is supported by reports that congenitally blind individuals have difficulties in creating allocentric memories^15,17^ and representations^14^ of three-dimensional space, in inferring spatial relationships between objects distributed in large-scale, unfamiliar environments^14^, in remembering distant spatial positions^89^ and spatial boundaries^90^, and in integrating egocentric information during movement through space^91^. All of these properties are functional attributes of the hippocampus that uses place cells^92–94^ and synaptic plasticity^61–63,95,96^ to create allocentric navigational maps^97–99^, and both integrate and store information about complex spatial associations^93,100–102^. Our finding that hippocampal synaptic plasticity is perpetually undermined in congenitally blind mice offers a functional explanation for these deficits: given that hippocampal LTP is an intrinsic cellular mechanism of the encoding and retention of spatial memory^96,103–105^, the limitations in allocentric encoding ability reported in congenitally blind individuals may derive from impoverished hippocampal LTP.

In conclusion, this study reveals that the process of cortical adaptation to loss of visual input is a dynamic process that is sustained throughout adulthood. The cortex does not revert back to its original state with time, rather it remains in a state that is similar to the critical period of postnatal cortical development. Furthermore, although many studies in rodents and humans have described how crossmodal perceptual ability and spatial acuity improve over time and reach levels that are equivalent to, or surpass those of individuals with intact senses, hippocampal synaptic plasticity is persistently debilitated following permanent sensory loss. This may explain, in part, why progressive sensory loss is accompanied by cognitive deficits.

Taken together these data indicate that the cortex and hippocampus are perpetually challenged by the absence of adequate sensory inflow and that intact multimodal sensory input is a prerequisite for effective experience-dependent information storage by the hippocampus.

## Supplementary Information

Supplementary Legends.

Supplementary Figure S1.

Supplementary Figure S2.

Supplementary Table S1.

Supplementary Table S2.

Supplementary Table S3.

Supplementary Table S4.

Supplementary Table S5.

## Data Availability

The imaging data will be publicly available online (https://doi.org/10.12751/g-node.ne87eh) and from the authors on reasonable request upon publication.
