# Supplementary material for: Lifelong changes of neurotransmitter receptor expression and debilitation of hippocampal synaptic plasticity following early postnatal blindness
Source: Sci Rep. 2022 Jun 1;12:9142. doi: 10.1038/s41598-022-13127-y (PMC9160005; doi:10.1038/s41598-022-13127-y)
Supplement: Supplementary file 1 — Supplementary Legends. [file 41598_2022_13127_MOESM1_ESM.docx]

**Supplementary figure S1. Comparison of input-output curves between CBA/CaOlaHsd and CBA/J mice at different ages.**

(A-H) A comparison of input-output strength between CBA/CaOlaHsd and CBA/J revealed no significant differences at (A) 3 months, (B) 4 months, (C) 5 months, (D) 6 months, (E) 9 months, (F) 10 months, (G) 11 months and (F) 12 months.

**Supplementary figure S2. Summary of changes in short-term and long-term potentiation between CBA/CaOlaHsd and CBA/J mice at ages.**

(A) Comparison of short-term (1-3h) and long-term (3-24h) plasticity between CBA/CaOlaHsd (blue bars) and CBA/J (green bars) is shown for 3, 4, 5 and 6 months of age. Black circles show individual responses.

(B) Comparison of short-term (1-3h) and long-term (3-24h) plasticity between CBA/CaOlaHsd (blue bars) and CBA/J (green bars) is shown for 9, 10, 11 and 12 months of age. Black circles show individual responses.
