# Supplementary figures and images for: Lifelong changes of neurotransmitter receptor expression and debilitation of hippocampal synaptic plasticity following early postnatal blindness
Source: Sci Rep. 2022 Jun 1;12:9142. doi: 10.1038/s41598-022-13127-y (PMC9160005; doi:10.1038/s41598-022-13127-y)

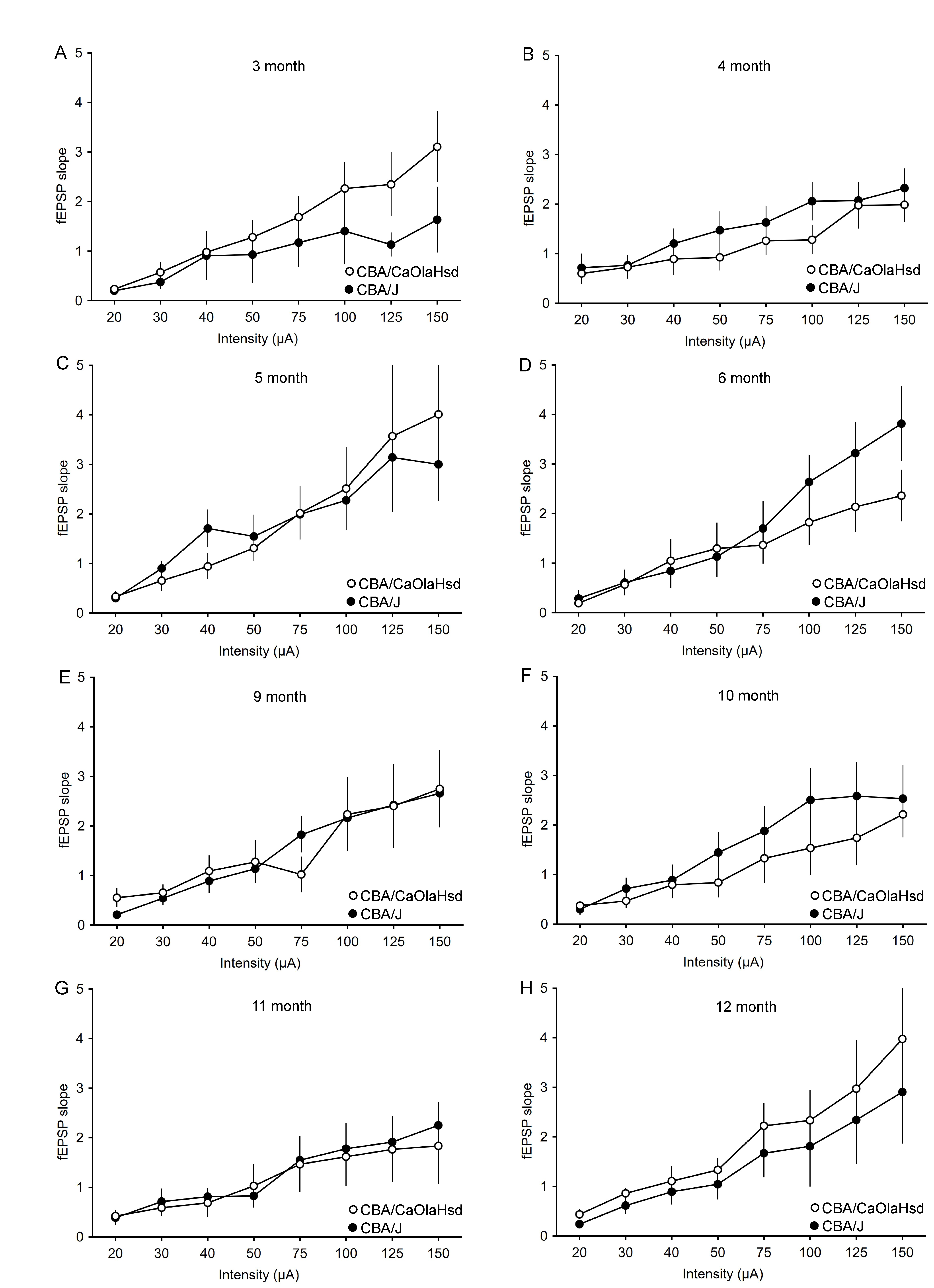

Supplement: Supplementary file 2 — Supplementary Figure S1. [file 41598_2022_13127_MOESM2_ESM.tif]

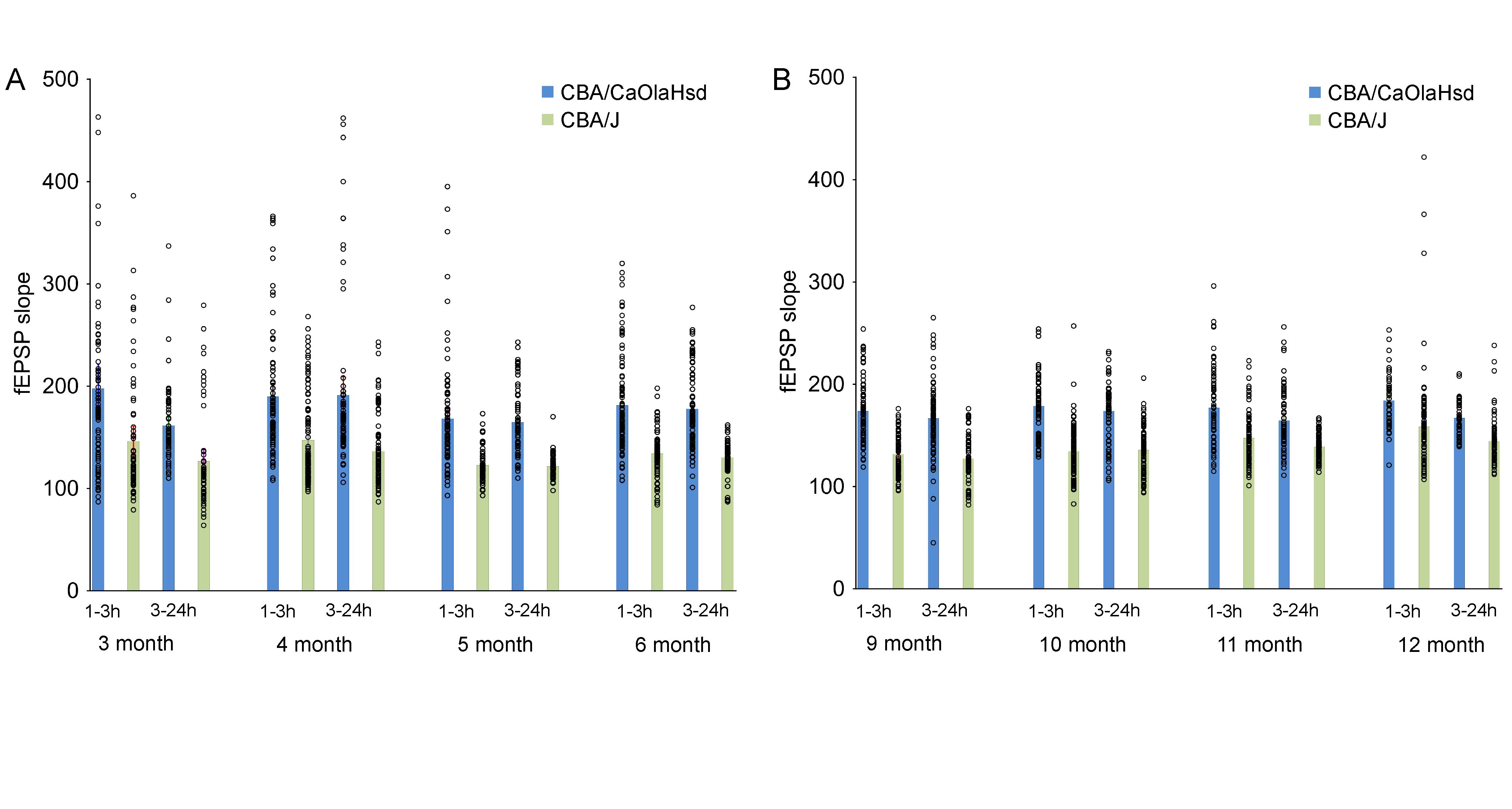

Supplement: Supplementary file 3 — Supplementary Figure S2. [file 41598_2022_13127_MOESM3_ESM.tif]
