# Supplementary material for: Lifelong changes of neurotransmitter receptor expression and debilitation of hippocampal synaptic plasticity following early postnatal blindness
Source: Sci Rep. 2022 Jun 1;12:9142. doi: 10.1038/s41598-022-13127-y (PMC9160005; doi:10.1038/s41598-022-13127-y)
Supplement: Supplementary file 4 — Supplementary Table S1. [file 41598_2022_13127_MOESM4_ESM.docx]

**Supplementary Table S1: Comparison of input-output curves of CBA/CaOlaHsd and CBA/J mice at different ages.**

A comparison of basal synaptic plasticity revealed no significant differences between CBA/CaOlaHsd and CBA/J mice at 3, 4, 5, 6, 9, 10, 11 and 12 months of age.

| **Month** | **Main effect** | **Interaction effect** |
| --- | --- | --- |
| 3 | F(1,11) = 1,22; p = 0,29 | F(7,77) = 1,80; p = 0,10 |
| 4 | F(1,14) = 0,93; p = 0,35 | F(7,98) = 0,91; p = 0,51 |
| 5 | F(1,10) = 0,01; p = 0,94 | F(7,70) = 0,34; p = 0,93 |
| 6 | F(1,11) = 2,48; p = 0,14 | F(7,77) = 1,91; p = 0,08 |
| 9 | F(1,14) = 0,001; p = 0,97 | F(7,98) = 0,63; p = 0,73 |
| 10 | F(1,12) = 1,04; p = 0,33 | F(7,84) = 1,49; p = 0,18 |
| 11 | F(1,11) = 0,04; p = 0,84 | F(7,77) = 0,23; p = 0,98 |
| 12 | F(1,9) = 0,61; p = 0,45 | F(7,63) = 0,37; p = 0,92 |
