# Supplementary material for: Lifelong changes of neurotransmitter receptor expression and debilitation of hippocampal synaptic plasticity following early postnatal blindness
Source: Sci Rep. 2022 Jun 1;12:9142. doi: 10.1038/s41598-022-13127-y (PMC9160005; doi:10.1038/s41598-022-13127-y)
Supplement: Supplementary file 5 — Supplementary Table S2. [file 41598_2022_13127_MOESM5_ESM.docx]

**Supplementary Table S2. Summary of the results of statistical analysis of receptor expression in blind (CBA/J) mice at 8 and 12 months postnatally, compared to healthy (CBA/CaOlaHsd) mice.** GABA receptor, NMDAR subunit and mGlu receptor-expression in the cortex and hippocampus of CBA/J mice were compared to expression levels in CBA/CaOlaHsd mice at 8 and 12 months of age. Optical density values as well as the significance (p) values, as determined by multifactorial analysis of variance followed by a post hoc Duncan’s test, are reported.

Piriform cortex (PiC), somatosensory cortex (SC), posterior parietal cortex (PPtA), visual cortex (VC), auditory cortex (AuC).

| **Receptor** | **Area** | **CBA/J** | **CBA/CaOlaHsd** | **p** | **n** |
| --- | --- | --- | --- | --- | --- |
|  |  |  |  |  |  |
| **8 Months** |  |  |  |  |  |
| **GluN1** | **PiC** | 13.84 ± 0.88 | 30.27 ± 2.30 | <0.001 | 5 |
|  | **SC** | 13.19 ± 0.65 | 29.76 ± 2.19 | <0.001 | 6 |
|  | **PPtA** | 13.84 ± 0.83 | 33.18 ± 1.96 | <0.001 | 6 |
|  | **VC** | 11.38 ± 0.81 | 31.24 ± 1.94 | <0.001 | 6 |
|  | **AuC** | 12.73 ± 0.98 | 35.74 ± 3.36 | <0.001 | 6 |
|  | **DG** | 11.82 ± 1.01 | 36.48 ± 3.27 | <0.001 | 6 |
|  | **CA1** | 18.09 ± 1.18 | 49.23 ± 4.25 | <0.001 | 6 |
|  | **CA3** | 21.78 ± 1.19 | 48.84 ± 3.52 | <0.001 | 6 |
|  | **CA4** | 12.64 ± 1.21 | 33.60 ± 2.69 | <0.001 | 6 |
| **GluN2A** | **PiC** | 20.56 ± 0.92 | 26.77 ± 1.24 | <0.001 | 5 |
|  | **SC** | 21.35 ± 0.66 | 28.06 ± 1.04 | <0.001 | 6 |
|  | **PPtA** | 18.81 ± 0.77 | 25.45 ± 1.53 | <0.001 | 6 |
|  | **VC** | 18.41 ± 0.92 | 24.48 ± 0.88 | <0.001 | 6 |
|  | **AuC** | 18.94 ± 0.78 | 25.07 ± 1.49 | <0.001 | 6 |
|  | **DG** | 14.27 ± 0.78 | 18.78 ± 0.79 | <0.01 | 6 |
|  | **CA3** | 22.56 ± 0.89 | 27.61 ± 0.99 | <0.001 | 6 |
|  | **CA4** | 14.53 ± 0.98 | 20.84 ± 0.92 | <0.001 | 6 |
| **mGlu1** | **PiC** | 40.95 ± 2.38 | 48.10 ± 3.37 | <0.05 | 5 |
|  | **SC** | 42.34 ± 1.45 | 49.15 ± 2.30 | <0.05 | 6 |
|  | **VC** | 39.73 ± 1.48 | 47.34 ± 3.56 | <0.05 | 6 |
|  | **AuC** | 42.92 ± 1.52 | 50.02 ± 2.76 | <0.05 | 6 |
| **mGlu2/3** | **SC** | 35.49 ± 0.97 | 42.16 ± 1.44 | <0.01 | 5 |
|  | **PPtA** | 35.81 ± 1.36 | 46.14 ± 1.61 | <0.001 | 6 |
|  | **VC** | 33.33 ± 0.89 | 42.28 ± 1.38 | <0.001 | 6 |
|  | **AuC** | 34.56 ± 1.40 | 41.54 ± 2.36 | <0.01 | 6 |
|  | **CA3** | 15.51 ± 0.74 | 20.88 ± 1.14 | <0.01 | 6 |
|  | **CA4** | 20.89 ± 0.91 | 25.85 ± 1.13 | <0.05 | 6 |
| **GABA_A_** | **CA1** | 64.80 ± 2.75 | 80.68 ± 3.72 | <0.05 | 4 |
| **GABA_B_** | **CA1** | 30.36 ± 2.71 | 45.75 ± 1.90 | <0.01 | 4 |
|  | **CA3** | 45.02 ± 3.35 | 57.57 ± 2.93 | <0.05 | 4 |
| **12 Months** |  |  |  |  |  |
| **GluN1** | **PiC** | 13.70 ± 0.80 | 31.64 ± 1.00 | <0.001 | 6 |
|  | **SC** | 14.96 ± 0.91 | 30.85 ± 0.82 | <0.001 | 6 |
|  | **PPtA** | 14.97 ± 1.12 | 37.69 ± 0.94     \| - \| \| --- \| \| - \| \| - \| \| **↓** \|   0 | <0.001 | 6 |
|  | **VC** | 14.52 ± 1.09 | 33.21 ± 0.92 | <0.001 | 6 |
|  | **AuC** | 16.81 ± 1.00 | 38.54 ± 1.35 | <0.001 | 6 |
|  | **DG** | 14.39 ± 0.89 | 34.43 ± 1.44 | <0.001 | 6 |
|  | **CA1** | 21.23 ± 1.10 | 47.30 ± 1.39 | <0.001 | 6 |
|  | **CA3** | 22.75 ± 1.02 | 46.22 ± 1.12 | <0.001 | 6 |
|  | **CA4** | 13.37 ± 0.93 | 30.39 ± 0.80 | <0.001 | 6 |
| **GluN2A** | **SC** | 30.15 ± 0.98 | 36.51 ± 1.38 | <0.01 | 6 |
|  | **VC** | 31.46 ± 1.08 | 35.99 ± 2.64 | <0.05 | 6 |
|  | **AuC** | 29.68 ± 0.89 | 36.37 ± 1.40 | <0.05 | 6 |
|  | **CA3** | 31.47 ± 0.76 | 39.21 ± 1.60 | <0.001 | 6 |
|  | **CA4** | 23.80 ± 0.65 | 28.17 ± 1.19 | <0.05 | 5 |
| **GluN2B** | **PiC** | 34.39 ± 1.60 | 27.41 ± 2.08 | <0.01 | 4 |
|  | **SC** | 39.77 ± 1.90 | 33.93 ± 1.59 | <0.05 | 5 |
|  | **PPtA** | 52.11 ± 2.18 | 42.92 ± 2.65 | <0.001 | 4 |
|  | **VC** | 48.45 ± 1.83 | 40.21 ± 1.33 | <0.001 | 5 |
| **mGlu1** | **PPtA** | 72.79 ± 1.95 | 62.00 ± 2.79 | <0.05 | 6 |
|  | **VC** | 72.24 ± 2.03 | 59.87 ± 2.78 | <0.01 | 6 |
|  | **DG** | 73.80 ± 2.50 | 62.94 ± 3.60 | <0.05 | 6 |
|  | **CA1** | 73.14 ± 2.66 | 60.67 ± 2.82 | <0.01 | 6 |
|  | **CA3** | 77.45 ± 2.14 | 67.07 ± 3.39 | <0.05 | 6 |
|  | **CA4** | 71.03 ± 2.38 | 59.83 ± 3.81 | <0.05 | 6 |
| **GABA_A_** | **PiC** | 81.11 ± 4.52 | 94.94 ± 3.47 | <0.05 | 5 |
|  | **PPtA** | 72.05 ± 5.87 | 88.46 ± 3.61 | <0.01 | 6 |
|  | **VC** | 75.49 ± 4.18 | 92.18 ± 3.86 | <0.01 | 6 |
|  | **AuC** | 84.28 ± 3.69 | 98.27 ± 5.43 | <0.05 | 6 |
|  | **DG** | 74.39 ± 3.42 | 87.68 ± 3.20 | <0.05 | 6 |
|  | **CA1** | 65.60 ± 3.58 | 84.60 ± 3.14 | <0.01 | 6 |
| **GABA_B_** | **AuC** | 30.71 ± 1.34 | 37.20 ± 1.72 | <0.05 | 6 |
|  | **CA1** | 32.04 ± 1.59 | 39.34 ± 1.75 | <0.05 | 6 |
