# Supplementary material for: Lifelong changes of neurotransmitter receptor expression and debilitation of hippocampal synaptic plasticity following early postnatal blindness
Source: Sci Rep. 2022 Jun 1;12:9142. doi: 10.1038/s41598-022-13127-y (PMC9160005; doi:10.1038/s41598-022-13127-y)
Supplement: Supplementary file 6 — Supplementary Table S3. [file 41598_2022_13127_MOESM6_ESM.docx]

**Supplementary Table S3. Overview of significant changes in receptor expression in 2-12 month old CBA/J** **compared to CBA/CaOlaHsd mice.**

GABA receptor, NMDAR subunit and mGlu receptor-expression in the cortex and hippocampus of CBA/J mice were compared to expression levels in CBA/CaOlaHsd mice at 2, 4, 8 and 12 months of age.

A significant decrease, or increase, in receptor expression (i.e. at least p<0.05) is signified by a downwards-pointing or an upwards-pointing arrow, respectively. A dash signifies an absence of significant effect. Detailed results for GABA receptor and GluN subunit expression in the 2-4 month old age-groups were reported by us previously^7,8^, but are included here to provide a comprehensive overview.

Piriform cortex (PiC), somatosensory cortex (SC), posterior parietal cortex (PPtA), visual cortex (VC), auditory cortex (AuC), dentate gyrus (DG), CA1 region, CA3 region and CA4 region.

|  | **CBA/J vs. CBA/CaOlaHsd** | | | | | | | | |
| --- | --- | --- | --- | --- | --- | --- | --- | --- | --- |
| **GABA_A_** | PiC | S1 | PC | V1 | Au1 | CA1 | CA3 | CA4 | DG |
| 2 months | - | - | - | - | - | - | - | - | - |
| 4 months | - | **↓** | **↓** | - | - | **↓** | - | - | **↓** |
| 8 months | - | - | - | - | - | **↓** | - | - | - |
| 12 months | **↓** | - | **↓** | **↓** | **↓** | **↓** | - | - | **↓** |
| **GABA_B_** | PiC | S1 | PC | V1 | Au1 | CA1 | CA3 | CA4 | DG |
| 2 months | - | - | - | - | - | - | - | - | - |
| 4 months | **↑** | - | - | - | - | - | - | - | - |
| 8 months | - | - | - | - | - | **↓** | **↓** | - | - |
| 12 months | - | - | - | - | **↓** | **↓** | - | - | - |
| **GluN1** | PiC | S1 | PC | V1 | Au1 | CA1 | CA3 | CA4 | DG |
| 2 months | - | - | - | - | - | - | - | - | - |
| 4 months | - | - | - | - | - | - | - | - | - |
| 8 months | **↓** | **↓** | **↓** | **↓** | **↓** | **↓** | **↓** | **↓** | **↓** |
| 12 months | **↓** | **↓** | **↓** | **↓** | **↓** | **↓** | **↓** | **↓** | **↓** |
| **GluN2A** | PiC | S1 | PC | V1 | Au1 | CA1 | CA3 | CA4 | DG |
| 2 months | - | - | - | - | - | - | - | - | - |
| 4 months | - | - | - | - | - | - | - | - | - |
| 8 months | **↓** | **↓** | **↓** | **↓** | **↓** | - | **↓** | **↓** | **↓** |
| 12 months | - | **↓** | - | **↓** | **↓** | - | **↓** | **↓** | - |
| **GluN2B** | PiC | S1 | PC | V1 | Au1 | CA1 | CA3 | CA4 | DG |
| 2 months | - | - | - | - | - | - | - | **↑** | - |
| 4 months | **↑** | **↑** | **↑** | **↑** | **↑** | **↑** | **↑** | **↑** | **↑** |
| 8 months | - | - | - | - | - | - | - | - | - |
| 12 months | **↑** | **↑** | **↑** | **↑** | - | - | - | - | - |
| **mGlu1** | PiC | S1 | PC | V1 | Au1 | CA1 | CA3 | CA4 | DG |
| 2 months | - | - | - | - | - | - | - | - | - |
| 4 months | - | **↑** | - | - | - | - | **↑** | - | - |
| 8 months | **↓** | **↓** | - | **↓** | **↓** | - | - | - | - |
| 12 months | - | - | **↑** | **↑** | - | **↑** | **↑** | **↑** | **↑** |
| **mGlu2/3** | PiC | S1 | PC | V1 | Au1 | CA1 | CA3 | CA4 | DG |
| 2 months | - | - | - | - | - | - | - | - | - |
| 4 months | **↑** | - | - | - | - | - | - | - | - |
| 8 months | - | **↓** | **↓** | **↓** | **↓** | - | **↓** | **↓** | - |
| 12 months | - | - | - | - | - | - | - | - | - |
| **mGlu5** | PiC | S1 | PC | V1 | Au1 | CA1 | CA3 | CA4 | DG |
| 2 months | - | - | - | - | - | - | - | - | - |
| 4 months | - | - | - | - | - | - | - | - | - |
| 8 months | - | - | - | - | - | - | - | - | - |
| 12 months | - | - | - | - | - | - | - | - | - |
