# Supplementary material for: Lifelong changes of neurotransmitter receptor expression and debilitation of hippocampal synaptic plasticity following early postnatal blindness
Source: Sci Rep. 2022 Jun 1;12:9142. doi: 10.1038/s41598-022-13127-y (PMC9160005; doi:10.1038/s41598-022-13127-y)
Supplement: Supplementary file 7 — Supplementary Table S4. [file 41598_2022_13127_MOESM7_ESM.docx]

**Supplementary Table S4:** **Statistical comparison of synaptic plasticity across the ages of CBA/CaOlaHsd mice.**

Long-term potentiation evoked in CBA/CaOlaHsd mice showed no significant changes when compared at all ages under examination. Significant effects are shown in red.

| **Months** | **Main effect** | **Interaction effect** |
| --- | --- | --- |
| 3 – 4 | F(1,12) = 0,1; p = 0,76 | F(22,264) = 0,76; p = 0,77 |
| 3 – 5 | F(1,12) = 0,25; p = 0,63 | F(22,264) = 0,98; p = 0,49 |
| 3 – 6 | F(1,11) = 0,35; p = 0,57 | F(22,242) = 0,85; p = 0,66 |
| 3 – 9 | F(1,13) = 0,18; p = 0,68 | F(22,286) = 1,13; p = 0,32 |
| 3 – 10 | F(1,12) = 0,023; p = 0,88 | F(22,264) = 1,05; p = 0,41 |
| 3 – 11 | F(1,11) = 0,11; p = 0,75 | F(22,242) = 0,67; p = 0,87 |
| 3 – 12 | F(1,9) = 0,02; p = 0,90 | F(22,198) = 0,38; p = 1,0 |
|  |  |  |
| 4 – 5 | F(1,12) = 0,84; p = 0,38 | F(22,264) = 0,39; n = 0,99 |
| 4 – 6 | F(1,11) = 1,06; p = 0,33 | F(22,242) = 0,25; n = 1,0 |
| 4 – 9 | F(1,13) = 0,82; p = 0,38 | (F22,286) = 0,37; n = 1,0 |
| 4 – 10 | F(1,12) = 0,34; p = 0,57 | F(22,264) = 0,40; n = 0,99 |
| 4 – 11 | F(1,11) = 0,56; p = 0,47 | F(22,242) = 0,45; n = 0,99 |
| 4 – 12 | F(1,9) = 0,23; p = 0,64 | F(22,198) = 0,39; n = 1,0 |
|  |  |  |
| 5 – 6 | F(1,11) = 0,01; p = 0,91 | F(22,242) = 0,31; n = 1,0 |
| 5 – 9 | F(1,13) = 0,05; p = 0,82 | F(22,286) = 0,61; n = 0,92 |
| 5 – 10 | F(1,12) = 0,31; p = 0,59 | F(22,264) = 0,40; n = 0,99 |
| 5 – 11 | F(1,11) = 0,06; p = 0,82 | F(22,242) = 0,61; n = 0,92 |
| 5 – 12 | F(1,9) = 0,21; p = 0,66 | F(22,198) = 0,66; n = 0,87 |
|  |  |  |
| 6 – 9 | F(1,12) = 0,2; p = 0,67 | F(22,264) = 0,72; p = 0,82 |
| 6 – 10 | F(1,11) = 0,67; p = 0,43 | F(22,242) = 0,34; p = 1,0 |
| 6 – 11 | F(1,10) = 0,18; p = 0,68 | F(22,220) = 0,78; p = 0,75 |
| 6 – 12 | F(1,8) = 0,59; p = 0,46 | F(22,176) = 1,02; p = 0,44 |
|  |  |  |
| 9 – 10 | F(1,13) = 0,21; p = 0,65 | F(22,286) = 0,85, p = 0,67 |
| 9 – 11 | F(1,12) = 0,01; p = 0,96 | F(22,264) = 0,89; p = 0,61 |
| 9 – 12 | F(1,10) = 0,16; p = 0,70 | F(22,220) = 0,80; p = 0,72 |
|  |  |  |
| 10 – 11 | F(1,11) = 0,12; p = 0,74 | F(22,242) = 0,79; p = 0,73 |
| 10 – 12 | F(1,9) = 0,01; p = 0,98 | F(22,198) = 1,95; p < 0,05 |
|  |  |  |
| 11 – 12 | F(1,8) = 0,09; p = 0,78 | F(22,176) = 0,62; p = 0,91 |
