# Supplementary material for: Lifelong changes of neurotransmitter receptor expression and debilitation of hippocampal synaptic plasticity following early postnatal blindness
Source: Sci Rep. 2022 Jun 1;12:9142. doi: 10.1038/s41598-022-13127-y (PMC9160005; doi:10.1038/s41598-022-13127-y)
Supplement: Supplementary file 8 — Supplementary Table S5. [file 41598_2022_13127_MOESM8_ESM.docx]

**Supplementary Table S5: Statistical comparison of synaptic plasticity across the ages of CBA/J mice.**

Long-term potentiation evoked in CBA/J mice showed significant changes at 5 months when compared to 10, 11 and 12 months. The significant effects are shown in red.

| **Months** | **Main effect** | **Interaction effect** |
| --- | --- | --- |
| 3 – 4 | F(1,13) = 0,05; p = 0,82 | F(22,286) = 0,39; p = 0,99 |
| 3 – 5 | F(1,9) = 0,36; p = 0,56 | F(22,198) = 1,53; p = 0,07 |
| 3 – 6 | F(1,11) = 0,04; p = 0,85 | F(22,242) = 0,91; p = 0,58 |
| 3 – 9 | F(1,12) = 0,13; p = 0,72 | F(22,264) = 1,75; p < 0,05 |
| 3 – 10 | F(1,11) = 0,01; p = 0,92 | F(22,242) = 2,0; p < 0,01 |
| 3 – 11 | F(1,11) = 0,09; p = 0,77 | F(22,242) = 0,72; p = 0,82 |
| 3 – 12 | F(1,11) = 0,43; p = 0,53 | F(22,242) = 0,74; p = 0,80 |
|  |  |  |
| 4 – 5 | F(1,12) = 1,89; p = 0,19 | F(22,264) = 0,79; p = 0,74 |
| 4 – 6 | F(1,14) = 0,48; p = 0,50 | F(22,308) = 0,40; p = 0,99 |
| 4 – 9 | F(1,15) = 0,99; p = 0,34 | F(22,330) = 0,86; p = 0,65 |
| 4 – 10 | F(1,14) = 0,05; p = 0,83 | F(22,308) = 1,54; p = 0,06 |
| 4 – 11 | F(1,14) = 0,01; p = 0,91 | F(22,308) = 0,44; p = 0,99 |
| 4 – 12 | F(1,14) = 0,46; p = 0,51 | F(22,308) = 0,75; p = 0,78 |
|  |  |  |
| 5 – 6 | F(1,10) = 1,90; p = 0,20 | F(22,220) = 0,32; p = 1,0 |
| 5 – 9 | F(1,11) = 0,88; p = 0,37 | F(22,242) = 0,28; p = 1,0 |
| 5 – 10 | F(1,10) = 5,29; p < 0,05 | F(22,220) = 0,51; p = 0,97 |
| 5 – 11 | F(1,10) = 9,10; p < 0,05 | F(22,220) = 0,95; p = 0,52 |
| 5 – 12 | F(1,10) = 7,0; p < 0,05 | F(22,220) = 0,97; p = 0,51 |
|  |  |  |
| 6 – 9 | F(1,13) = 0,15; p = 0,71 | F(22,286) = 0,26; p = 1,0 |
| 6 – 10 | F(1,12) = 0,56; p = 0,47 | F(22,264) = 0,90; p = 0,60 |
| 6 – 11 | F(1,12) = 1,66; p = 0,22 | F(22,264) = 0,40; p = 0,99 |
| 6 – 12 | F(1,12) = 2,96; p = 0,11 | F(22,264) = 0,96; p = 0,51 |
|  |  |  |
| 9 – 10 | F(1,13) = 1,31; p = 0,27 | F(22,286) = 1,1; p = 0,35 |
| 9 – 11 | F(1,13) = 2,89; p = 0,11 | F(22,286) = 1,0; p = 0,45 |
| 9 – 12 | F(1,13) = 4,37; p = 0,06 | F(22,286) = 1,20; p = 0,25 |
|  |  |  |
| 10 – 11 | F(1,12) = 0,30; p = 0,60 | F(22,264) = 1,06; p = 0,40 |
| 10 – 12 | F(1,12) = 1,37; p = 0,26 | F(22,264) = 1,24; p = 0,21 |
|  |  |  |
| 11 – 12 | F(1,12) = 0,61; p = 0,45 | F(22,264) = 0,67; p = 0,87 |
